# Combined therapy with cyclophosphamide and DNA preparation inhibits the tumor growth in mice

**DOI:** 10.1186/1479-0556-7-12

**Published:** 2009-08-14

**Authors:** Ekaterina A Alyamkina, Evgenia V Dolgova, Anastasia S Likhacheva, Vladimir A Rogachev, Tamara E Sebeleva, Valeriy P Nikolin, Nelly A Popova, Konstantin E Orishchenko, Dmitriy N Strunkin, Elena R Chernykh, Stanislav N Zagrebelniy, Sergei S Bogachev, Mikhail A Shurdov

**Affiliations:** 1 Novosibirsk State University, Novosibirsk, Russia; 2 Institute of Cytology and Genetics, Siberian Branch, Russian Academy of Sciences, Novosibirsk, Russia; 3 Municipal Hospital, Oncology Department, Novosibirsk, Russia; 4 Institute of Clinical Immunology, Siberian Branch, Russian Academy of Medical Sciences, Novosibirsk, Russia; 5 LLC Panagen, Gorno-Altaisk, Russia

## Abstract

**Background:**

When cyclophosphamide and preparations of fragmented exogenous genomic double stranded DNA were administered in sequence, the regressive effect on the tumor was synergic: this combined treatment had a more pronounced effect than cyclophosphamide alone. Our further studies demonstrated that exogenous DNA stimulated the maturation and specific activities of dendritic cells. This suggests that cyclophosphamide, combined with DNA, leads to an immune response to the tumors that were grafted into the subjects post treatment.

**Methods:**

Three-month old CBA/Lac mice were used in the experiments. The mice were injected with cyclosphamide (200 mkg per 1 kg body weight) and genomic DNA (of human, mouse or salmon sperm origin). The DNA was administered intraperitoneally or subcutaneously. After 23 to 60 days, one million tumor cells were intramuscularly grafted into the mice. In the final experiment, the mice were pre-immunized by subcutaneous injections of 20 million repeatedly thawed and frozen tumor cells. Changes in tumor growth were determined by multiplying the three perpendicular diameters (measured by caliper). Students' t-tests were used to determine the difference between tumor growth and average survival rate between the mouse groups and the controls.

**Results:**

An analysis of varying treatments with cyclophosphamide and exogenous DNA, followed by tumor grafting, provided evidence that this combined treatment had an immunizing effect. This inhibitory effect in mice was analyzed in an experiment with the classical immunization of a tumor homogenate. The strongest inhibitory action on a transplanted graft was created through the following steps: cyclophosphamide at 200 mg/kg of body weight administered as a pretreatment; 6 mg fragmented exogenous DNA administered over the course of 3 days; tumor homogenate grafted 10 days following the final DNA injection.

**Conclusion:**

Fragmented exogenous DNA injected with cyclophosphamide inhibits the growth of tumors that are grafted to mice after this combined treatment.

## Background

There is considerable interest in immunomodulatory oligonucleotides (IMOs) that either contain CpG motifs or have a phosphorothioate backbone [1]. Experimental data indicated that these DNA, when administered systemically, were able to induce the adaptive immune response. This property of IMOs is widely discussed in terms of its use for cancer immunotherapy [2-6].

IMOs act as a stimulant on immunocompetent T-lymphocytes, natural killer cells, macrophages, and dendritic cells (DCs). DCs are the primary target. IMOs, as an inducer of DC immunocompetency (depending on conditions), can exert both anticancer and suppressive influences. DCs treated with specific IMOs affect the direction of differentiation in naive CD4+ CD25- T-cells [7,8]. There is experimental evidence indicating that the immunogenic properties of IMOs are due to their effect on the Toll-like receptors (TLRs) detected in large quantities in plasmatic DCs and macrophages [9-11]. TLR9s are the pattern-recognizing receptors that initiate the innate and adaptive immunity. Interaction of DC TLR9 with a specific IMO ligand is the first and crucial step in activating the DC's ability to induce a biological anticancer effect; subsequently, the synthesis and secretion of main cytokines and T-lymphocyte differentiation take place. When the T8+ pathway is activated, DCs efficiently present antigens (AGs) and tumor AGs to T-cytotoxic lymphocytes in order to stimulate their proliferation. This leads to the formation of an anti-tumor adaptive immune response.

The regression stimulated by this cytostatic treatment synergize with subsequent IMO injections [4]. The anti-tumor activities of specific nucleotides, when administered immediately after cytostatic treatments, are considerably augmented. It is imperative to strictly adhere to the administration of cytostatics (including cyclophosphamide (CP)), followed by IMOs, in order to synergize the components and increase their efficacy as a cancer treatment.

The synergy of these components could stem from the decreased number of regulatory T-lymphocytes (Tregs). This decrease suppresses the Tregs' adaptive immunity, and delays their development (in comparison to CD8+ T-lymphocytes after myelosupression under cytostatic effect). Another possible explanation for the synergistics is that cytostatics enhance the immune response to tumor AGs (thus altering their immunogenicity).

Inhibition of the Tregs antitumor response is presumably a major obstacle to the success of tumor vaccinations and immunotherapy [4,12]. Based on clinical trials, it may be assumed that the efficacy of antitumor IMO therapy may be boosted by a pre-inactivation of Tregs. Treatments with cytostatics at therapeutic doses kill lymphocytes of all types, irrespective of their properties. The results of many studies provide evidence that Tregs may have a greater sensitivity to cytostatics than normal T-cells [13-20]. It thus appears that chemotherapy can selectively and strongly alter Tregs, while sparing the viability of T-cytotoxic lymphocytes, which are the determinants of the high anti-tumor efficiency of this therapy [17,21,22]. Tumor microenvironments harbor the activity of Tregs, suppressing the immune effect on tumor cells and thus protecting the tumor from immune regression. In such a case, chemotherapy not only decreases the number of Tregs, but also abolishes their defense function [14,20,23-25]. The stripped nude tumor is rendered susceptible to the effect of the innate and adaptive immunity induced by IMOs. Tumor microenvironments actually change during sparing treatment with cytostatics. DCs become activated and form a T-cytotoxic response to the tumor (which had previously escaped immune surveillance [4]).

Our previous studies established that not only IMOs, in combination with cytostatics, had a suppressive effect on tumor development; tumor growth was also significantly inhibited by a combined treatment of CP and human genomic double stranded DNA (dsDNA) fragmented to 200–6000 bp [26]. This combined treatment was much more effective than treatment with CP alone.

When exogenous DNA was used as a leukostimulator after CP-induced myelosuppression, tumors that were grafted post treatment were reduced. Combined treatment of CP and DNA was successful at strongly suppressing growth of tumor grafted before the treatment [26] and after it [this study].

Our further studies demonstrated that fragmented exogenous genomic dsDNA stimulates the maturation of DCs and activates their specific activity [unpublished data]. We suggest that treatment with CP and exogenous DNA leads to activation of the immune system.

In recent experiments, we tested regimens of CP and fragmented genomic DNA administration. We also followed the timeline of change in tumors that were grafted to mice (pre-treated or not with AGs). CP injections, in combination with subsequent fragmented genomic dsDNA treatments, provided evidence that this co-therapy had a pronounced antitumor effect on tumor grafts.

## Methods

### Animals

Three-month old CBA/Lac mice that were bred at the animal facility of the Institute of Cytology and Genetics (the Siberian Branch of the Russian Academy of Sciences) were used in experiments. Mice in groups of 10 were housed in plastic cages. They had free access to food and water. All experiments were performed in accordance with protocols approved by the Animal Care and Use Committee of the Institute of Cytology and Genetics.

### Preparations of DNA

Human DNA preparations were isolated from the placentas of healthy women using a phenol-free method; this made it possible to obtain a genome that preserves the fragments that are *in vivo *associated with the nuclear matrix (scaffold) proteins. The DNA preparation did not contain histones and polysaccharides; it was endotoxin-free. Mouse DNA was isolated from a mixture of tissues (thymus, liver, kidneys, spleen) and salmon sperm DNA was isolated from salmon sperm. DNA was fragmented in an ultrasonic disintegrator at a frequency of 22 kHz, to obtain a mixture of DNA fragments with a size of 200 to 6000 bp. DNA preparations were dissolved in saline and stored at a temperature of -20°C.

### Mouse treatment regimens

**Figure 1:**
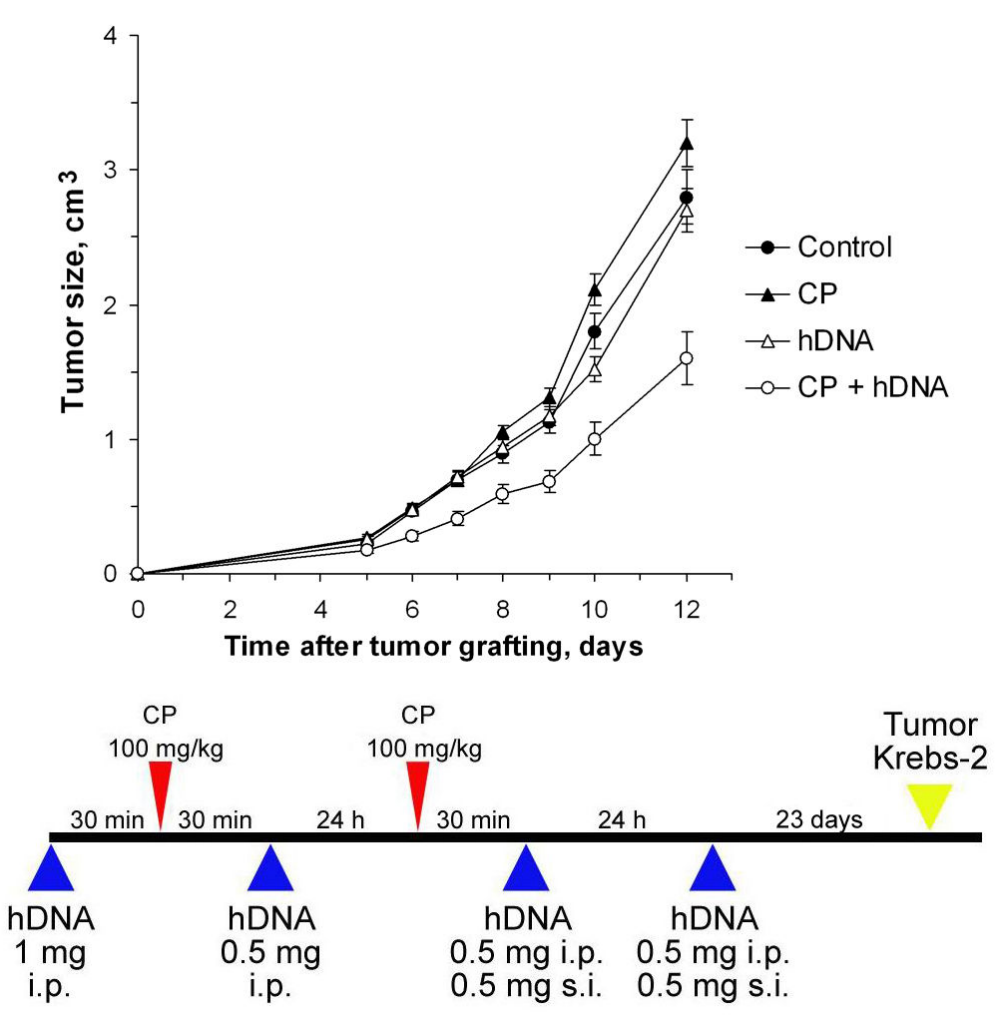
**Tumor growth (mean ± SEM) in mice treated with CP and human DNA, comparing the regimen shown below with the control (n = 10)**. Mice received two CP injections (100 mg per 1 kg body weight) at a daily interval; 0.5–1 mg human DNA was administered i.p. or s.c. to the mice. The control group was injected with saline. The additional control groups received either CP alone or DNA alone according to the regimen. Krebs-2 tumor cells were grafted i.m. 23 days after the last DNA administration.

The mouse treatment regimens are schematically represented in the figures that can be found in the Results section. CP (Veropharm, Russia) that was dissolved in saline was injected intraperitoneally (i.p.). Mice received either one CP injection (experiments 3–6) or two (experiments 1 and 2) on a daily interval. The total CP dose did not exceed 200 mg per 1 kg of body weight. This was followed by 3–12-fold administrations of 1 mkg – 2 mg DNA preparations (of human, mouse or salmon sperm origin) that were injected i.p. or subcutaneously (s.c.) into the backs of mice for 1–3 days. In experiments 1 and 2, mice were additionally i.p. injected with 1 mg DNA 30 min prior to the CP injection, and they received 0.5 mg DNA during the interval between the two CP injections (30–40 min after the first CP dose). The control groups in experiments 1 and 5 were treated with saline instead of DNA or CP. The control groups in experiment 1 were mice that received either CP alone or DNA alone according to the regimen given in Fig. 1. The control mice in experiment 5 were given CP 200 mg/kg two months before the tumors were grafted. Tumor cells were grafted 23 – 60 days after the last DNA administration. Groups of 6–10 mice were used for each experiment.

### Tumor models

We used Krebs-2 and lymphosarcoma (LS) tumors. The transplantable mouse LS was induced by V.I. Kaledin (Institute of Cytology and Genetics, Siberian Branch of the Russian Academy of Sciences, Novosibirsk, Russia) in CBA mice using nitrosomethylurea, transformed into an ascitic form, and maintained in this line. LSs are highly sensitive to the apoptosis induced by CP and several other alkylating agents. The Krebs-2 tumors were initially derived from mammary gland adenocarcinomas; they are mouse nonspecific and do not spread by metastases.

Tumor cells (1 × 10^6^) were grafted intramuscularly (i.m.) into the right hind thigh of the mice. Changes in tumor growth (cm^3^) were determined by multiplying the three perpendicular diameters (measured by caliper). These measurements were done 8–17 days after grafting.

### Immunization experiment

In experiment 6, 10 days after the mice that were injected with CP and human DNA were preimmunized with tumor AGs (by s.c. injection into the dorsal back of 20 × 10^6 ^repeatedly thawed-frozen Krebs-2 tumor cells), 1 × 10^6 ^Krebs-2 tumor cells were grafted i.m. into the right hind thigh of the mice. In this experiment, there were two additional control groups; one was immunized only, and the other was immunized after the CP injection.

### Statistical analysis

Students' t-tests were used to determine the significance of the differences in tumor growth, and average survival between the mouse groups and the controls. All results were expressed as mean ± SEM.

## Results

### Suppression of growth of experimental tumors in regimens of CP injection and exogenous dsDNA administration

We estimated the co-treatment effect of cytostatic CP, combined with a preparation of exogenous fragmented dsDNA, on the growth of experimental tumors in mice. At the early phase of the experiment, we chose the parameters for CP injections, exogenous DNA administrations, and tumor grafting with the following considerations.

The activating effect of exogenous DNA on the immune system was estimated first. For this reason, all treatments were done prior to tumor grafting. Moreover, we had established that the administration of exogenous dsDNA, 30–60 min before or after the CP injection, had no statistically significant effect on tumor growth suppression [26]. The retardation of the grafted tumor growth became conspicuous when the interval between DNA administration and CP injection was long (1–3 days after CP injection). Several administrations of exogenous dsDNA for 1–3 days after CP injections most efficiently suppressed tumor growth [26].

With the above parameters, we designed experiments for immune system activation in treated mice. The design included CP injections, administrations of exogenous DNA preparation and tumor grafting after different time intervals.

### Estimation of the effect of regimens of CP plus exogenous fragmented dsDNA on tumor growth

We proceeded to examine the effect of CP injections or fragmented human dsDNA administrations prior to grafting Krebs-2 tumors (Experiment 1). As shown in Fig. 1, the effect of only CP or DNA on the tumor was statistically insignificant (p > 0.05, n = 10).

**Figure 2:**
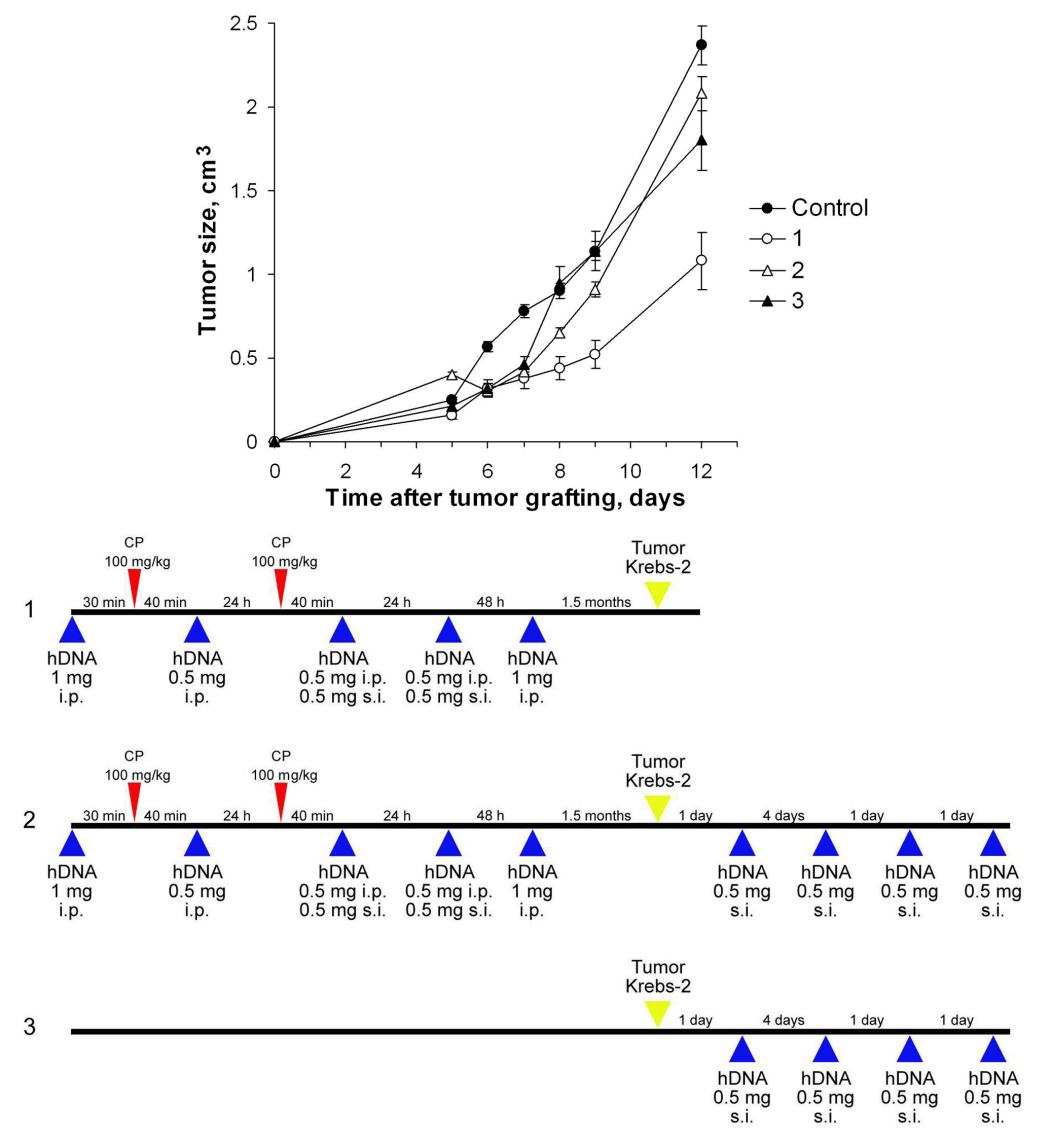
**Tumor growth (mean ± SEM) in mice treated with CP and human DNA, comparing the regimen shown below with the control (n = 10)**. Mice received two CP injections (100 mg per 1 kg body weight) on a daily interval; 0.5–1 mg human DNA were administered i.p. or s.c. to the mice. The control group was injected with saline. Krebs-2 tumor cells were grafted i.m. 1.5 month after the last DNA administration. The other group (2) received four human DNA s.c. injections after the tumor grafting. And the last group (3) didn't received CP but only four administrations of exogenous DNA preparations after the tumor grafting.

In our further experiments, we used versions of CP injections combined with administrations of fragmented exogenous DNA. Fig. 2 presents the three regimens for cytostatic injections and administrations of exogenous DNA (Experiment 2). The strongest suppressive effect on the grafted Krebs-2 tumor was created by Regimen 1 (p < 0.001, n = 10): CP was injected two times in combination with DNA (after defined intervals), and tumor were grafted 3 weeks – 1.5 months (the experiment was replicated several times) after the last DNA administration. The treatment protocol followed in Regimen 2 differed from Regimen 1 in that the mice received four additional exogenous DNA injections after the tumor grafting; this completely abolished the suppressive effect of the Regimen 1 therapy and induced the progression of the graft. We believe that the number and function of Tregs immune suppressors recovered by the time we started to repeatedly administer exogenous DNA. Injected exogenous DNA had already driven the adaptive response toward the Tregs phenotype; this led to suppression of the initially activated immune response and tumor progression.

Four administrations of exogenous DNA preparations according to Regimen 3 (after the tumor grafting only) had a weaker effect than Regimen 1; however, they had a significant (p < 0.05, n = 10) effect on Krebs-2 tumor growth in comparison with the controls.

**Figure 3:**
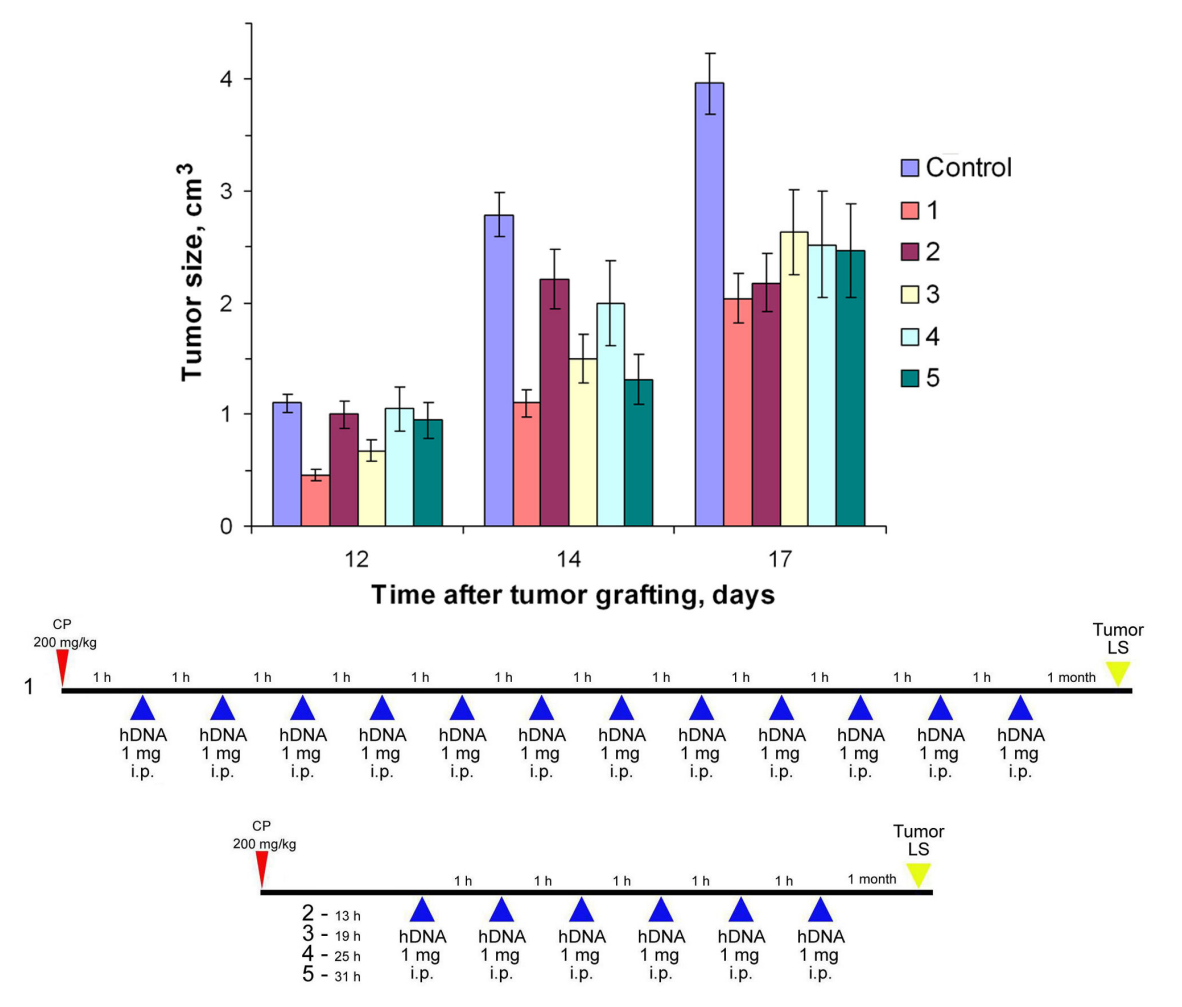
**Tumor growth (mean ± SEM) in mice treated with CP and human DNA, comparing the regimens shown below with the control (n = 6)**. Mice received CP injections (200 mg per 1 kg body weight); 1 (1), 13 (2), 19 (3), 25 (4), 31 (5) h after 1 mg DNA was administered i.p. every hour, 12 (1) or 6 (2–5) times. The control group was injected with saline. LS tumor cells were grafted i.m. 1 month after the last DNA administration.

18–30 h after systemic CP injections, interstrand crosslinks begin to repair from start to finish. These cross links are a result of human fragmented DNA presumably integrating extensively into the genome of the experimental mice. This integration was lethal for most mice [27]. To estimate how this effect may concern a synergic cooperation of the two agents, we performed Experiment 3 using a new regimen for combined treatment with cytostatic and DNA (Fig. 3). Mice received human DNA preparations every hour for 12 h after CP injections (Regimen 4) and hourly for 6 h, 13–18, 19–24, 25–30, and 31–36 h after CP injections (6 mice per group).

It was found that survival significantly improved (p < 0.05, n = 6) in groups 1 and 5 (mice that were treated with DNA1-13 and 31–36 h after the CP injection) compared with those of the control group (Table 1). It was also found that survival of group 4 (25–30 h) was insignificantly shorter (p > 0.05, n = 6: by 12%) than the control group. We believe that the reduced survival rate after the treatments during this interval was due to the extensive integration of exogenous DNA fragments into the genomes of treated mice, which uncoupled primary vital systems and developed diseases that lead to death.

**Table 1:** Average survival of mice in Experiment 3.

Group	Survival, days
Control	18.7 ± 0.8

1	22.1 ± 1.2

2	18.2 ± 1.4

3	19.0 ± 1.1

4	16.4 ± 0.2

5	24.2 ± 2.0

The values are means ± SEM (n = 6).

The increased survival rate of groups 1 and 5 can be explained through the timing of the repair mechanism: it did not start at the first time interval, but it was consummate at the second. Thus, exogenous DNA could not integrate, and DNA stimulated DCs and an increased immune response caused a statistically significant increase (p < 0.05, n = 6) in survival.

Regimen 4 was not substantially different from the general outline described in the beginning of the section. Its set of experiments resulted with the persistent suppression of grafted tumors. Its efficiency is comparable to that obtained with Regimen 1.

Using Regimen 4, we estimated the inhibitory effect of single and multiple hourly administrations of exogenous DNA preparations for 12 h after the CP injection (data not shown). Evidence indicated that multiple administrations of DNA preparation 0–12 h after the CP injection led to suppressed tumor growth. Vice versa, a single exogenous DNA administration at different times for 1–12 h after the CP injection had no suppressive effect on the growth of a grafted Krebs-2 tumor.

**Figure 4:**
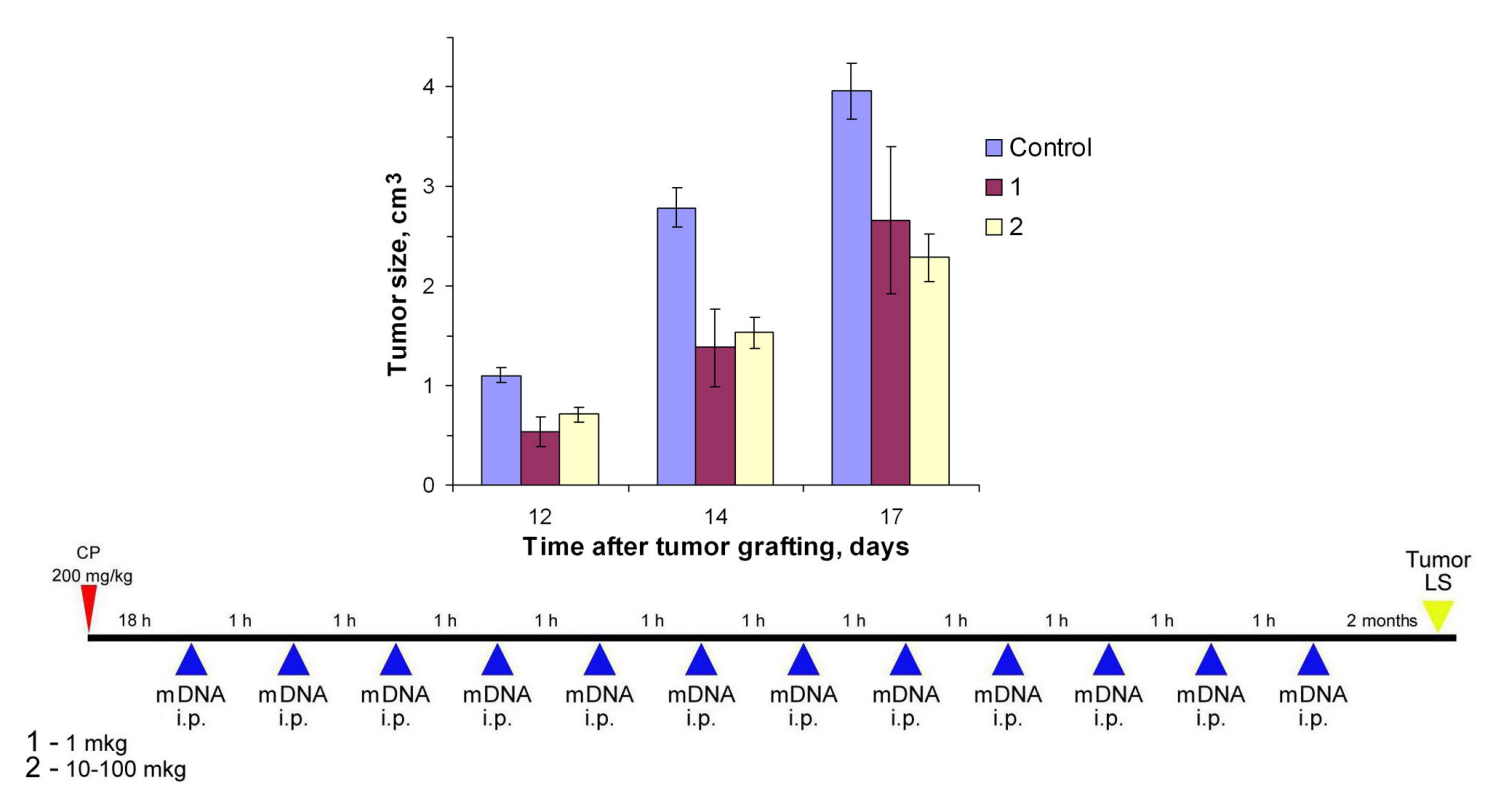
**Tumor growth (mean ± SEM) in mice treated with CP and different doses of mouse DNA, comparing the regimen shown below with the control (n = 7)**. Mice received CP injections (200 mg per 1 kg body weight); 18 h afterward 1 mkg (1) or 10–100 mkg (2) mouse DNA was administered i.p. every hour 12 times. The control group was injected with saline. LS tumor cells were grafted i.m. 2 months after the last DNA administration.

Analysis of a dose-dependent suppressive effect (with dsDNA preparations) provided evidence that 10–100 mkg was an efficient dose (p < 0.005, n = 7) to suppress tumor growth, while 1 mkg per mouse insignificantly suppressed tumor growth (p > 0.05, n = 7) (Experiment 4, Fig. 4). Overall, the preparations increased the average mice survival insignificantly (p > 0.05, n = 7) (Table 2). The DNA used in this experiment was allogenic, obtained from CBA mice.

**Table 2:** Average survival of mice in Experiment 4.

Group	Survival, days
Control	18.7 ± 0.8

1	25.0 ± 3.4

2	23.1 ± 2.0

The values are means ± SEM (n = 7).

### Estimation of the effect of exogenous DNA from different organisms on tumor growth on the background of CP therapy

**Figure 5:**
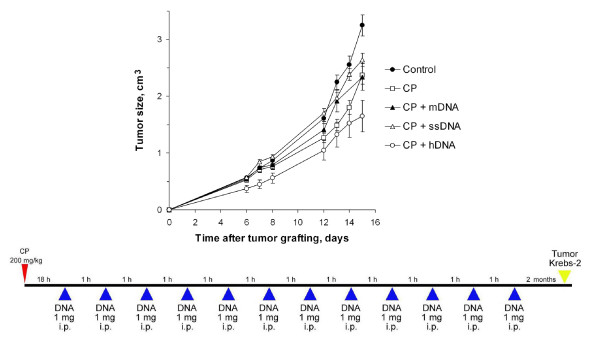
**Tumor growth (mean ± SEM) in mice treated with CP and DNA preparations human, mouse or salmon sperm origin, comparing the regimen shown below with the control (n = 6)**. Mice received CP injections (200 mg per 1 kg body weight); 18 h afterward saline ("CP") or 1 mg of genomic DNA preparations of mouse ("CP + mDNA"), salmon sperm ("CP + ssDNA") or human ("CP + hDNA") DNA were administered i.p. every hour for 12 times. The control group was injected with saline. Krebs-2 tumor cells were grafted i.m. two months after the last DNA administration.

In Experiment 5, we analyzed the effect of exogenous DNA based on its species origin (Fig. 5). Regimen 4 was chosen for obtaining estimates. The results showed that human xenogenic DNA combined with CP injections had the strongest statistically significant suppressive effect (p < 0.005, n = 6) on grafted tumor development, compared to allogenic mouse DNA (p < 0.05, n = 6) and distantly related DNA derived from salmon sperm (p < 0.05, n = 6).

### Estimation of immunization intensity for sequential treatment with cytostatic and exogenous genomic dsDNA

**Figure 6:**
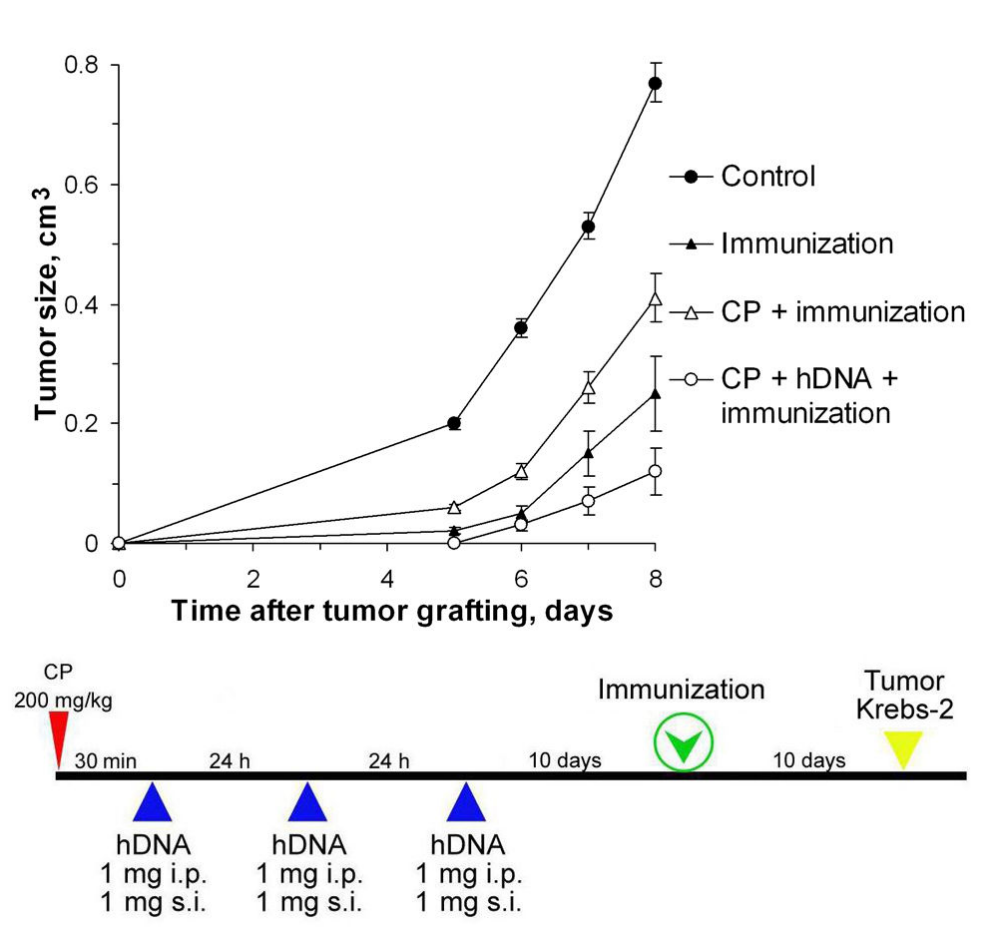
**Tumor growth (mean ± SEM) in mice treated with CP and human DNA, comparing the regimen shown below with the control (n = 10)**. Mice received CP injections (200 mg per 1 kg body weight); 30 min afterward and during the consecutive two days, mice were injected with human DNA, 1 mg i.p. and 1 mg s.c.. Mice were preimmunized with tumor AGs by s.c. injection into the dorsal back of 20 × 10^6 ^repeatedly thawed-frozen Krebs-2 tumor cells 10 days after the last DNA administration; 10 days after immunization 1 × 10^6 ^Krebs-2 tumor cells were grafted i.m. into the right hind thigh of mice. The control group was injected with saline. There were two additional control groups; one was immunized only, and the other was immunized after the CP injection.

In Experiment 6, mice were additionally immunized with a tumor cell homogenate after CP and exogenous DNA (Fig. 6). This co-treatment had the strongest suppressive effect on the grafted tumor in comparison to the control (p < 0.001, n = 10). The solitary immunizations and the immunizations with CP, without DNA, were weaker.

**Figure 7:**
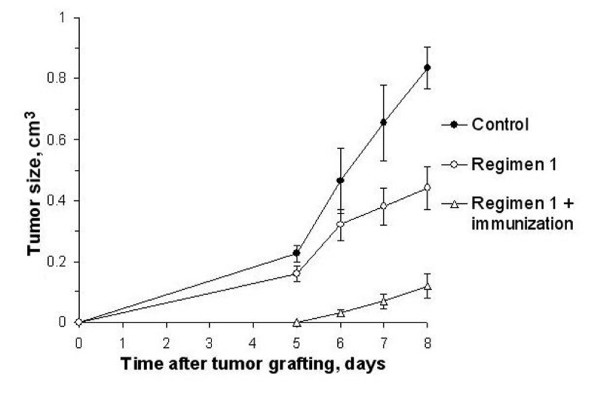
**Tumor growth (mean ± SEM) in mice treated according to Regimen 1 and additionally immunized (n = 10)**. The control group was injected with saline. In Regimen 1, mice received two CP injections (100 mg per 1 kg body weight) on a daily interval; 0.5–1 mg human DNA were administered i.p. or s.c. Krebs-2 tumor cells were grafted i.m. 1.5 month after the last DNA administration. Regimen 1 + immunization, mice received CP injection (200 mg per 1 kg body weight); 30 min afterward and during the consecutive two days, mice were injected with human DNA, 1 mg i.p. and 1 mg s.c.. Mice were pre-immunized with Krebs-2 tumor AGs by a s.c. injection 10 days after the last DNA administration; Krebs-2 tumor cells were grafted i.m. 10 days after immunization. Immunization enhanced the suppressive effect on tumor growth.

Comparing the volumes of Krebs-2 tumors grafted according to Regimen 1 (Experiment 2) with those immunized additionally in the interval between the last DNA administration and grafting (Experiment 6) demonstrated that immunization enhanced the suppressive effect on tumor growth (Fig. 7). The following regimen exerted the strongest inhibitory action on the graft: pre-treatment with CP at 200 mg/kg of body weight; fragmented exogenous DNA given for 3 days at a total dose of 6 mg; tumor homogenate injected 10 days after last DNA injection.

## Discussion

The present results evidence that exogenous DNA administered to experimental mice in combination with the cross linked cytostatic CP has an immunizing action and suppresses growth of a tumor that is grafted after this treatment. This means that CP/exogenous DNA co-treatments prepare the immune system to give a rapid specific immune response when tumor AGs arise. This co-treatment activates the immune system to acquire the ability to recognize tumor AGs and respond efficiently. The results of our concomitant study disclosed that this observed property of exogenous DNA is due to activation of DC maturation and a drive of the adaptive response toward cytotoxic T-cells [unpublished data].

Experiments designed to elucidate the synergic suppressive effect of cytostatics and immunomodulatory DNAs (which CpG DNA with a normal sugar phosphate backbone and oligonucleotides whose backbone sugar phosphorothioate belong to) are widely discussed. To our knowledge, all studies have attributed this synergic suppressive effect to the activation of both the innate immune and (more frequently) the adaptive immune response to the spreading tumor tissue. Differentiated suppression of Tregs and CD8+ T-cytotoxic lymphocytes under the effect of cytostatic, in the case of CP and IMOs (CpG) co-therapy, leads to activation of the innate and adaptive immunity.

Tumors induce the rapid capture of Tregs and Tregs-produced cytokines that inhibit the adaptive immunity [28-30]. CP creates and defines conditions for the differentiated suppression of T-cytotoxic and T-regulatory lymphocytes, and there exists an interval when the CD8+ T-cells: Tregs ratio becomes skewed by an order of two magnitudes in favor of T killer cells [14,15,17,18,23,28,31,32]. The difference in suppression degree and recovery rate between CD8+ lymphocytes and Tregs is important to cancer therapy. This is the time when the tumor becomes detectable by the non-supressed immune system. Tumor AGs are presented on DCs, and the surviving CD8+ T-lymphocytes (those not under the effect of cytokines produced by Tregs) kill cells of the developing tumor [23].

In the experimental studies, mice received CP after the tumors' stable growth. Tumor cells were left to die for some days after cytostatic treatment [33]. It was thought that at this time DCs absorbed apoptotic bodies of dead tumor cells or DNA of another kind as a result of lysed tumor cells. The adaptive immune response was simultaneously stimulated by IMOs via the CD8+ T-cell pathway; this led to active presentation of tumor AGs and the proliferation of lymphocytes [1,14,20,23-25,33-37].

In the studies, we used a novel regimen. Mice were first treated with a combination of CP and a human fragmented dsDNA preparation; this was followed by tumor grafting. With this treatment, the growth of many grafted tumor cells was substantially suppressed. To reiterate, our previous study had shown that the dsDNA preparation activates DCs ex vivo and induces their maturation and allostimulatory activity [unpublished data]; it is precisely this link of the immune system that activates tumor suppression.

In the experiments, doses of CP were therapeutic standard (200 mg/kg). The results clearly showed that the suppressive effect on tumor progression was manifested in all the experiments. This was due to activation of DCs by exogenous DNA, which in turn induced the adaptive immunity. Since CP at the applied doses completely eliminated both CD8+ T-cells and Tregs, exogenous DNA presumably activated DCs in such a way that just the T-cytotoxic adaptive (not the Treg suppressive) immune response was activated. This suggested that cytostatic and exogenous DNA combined treatments did not need a reduction in doses of CP for suppression of the two lymphocyte types; CP can be used at doses approved in modern practice; and the adaptive immune response is inducible at a defined time with exogenous dsDNA preparations.

Exogenous DNA activates the adaptive immune system 1–3 days after the injection of CP, resulting in suppression of the grafted tumor. We chose to graft the tumors 1–2 months after the last administration of the exogenous DNA preparations based on our experience. There may be other time intervals used to achieve a stronger suppressive action on tumors.

With the literary data taken into consideration, there are grounds to believe that pre-treatments with CP and exogenous DNA create an environment with effecter T-lymphocytes and DNA-activated DCs. This is after CP-induced myelosuppression is affected through the proliferation induction of cytotoxic, not suppressor, T-lymphocytes.

## Conclusion

Injections of fragmented exogenous DNA, combined with CP, inhibit the growth of tumors that are grafted to mice post treatment. It is assumed that this observed property of exogenous DNA is due to activation of DC maturation and a drive of the adaptive response toward cytotoxic T-cells.

## Competing interests

The authors declare that they have no competing interests.

## Authors' contributions

EAA carried out the mice experiments and performed the statistical analysis. EVD carried out the mice experiments and performed the statistical analysis. ASL carried out the mice experiments and drafted the manuscript. VAR participated in the design of the study. TES participated in the study design and helped with drafting the manuscript. VPN carried out the mice experiments, performed the analysis, and interpreted the data. NAP participated in the design of the study and performed the statistical analysis. KEO participated in the design of the study. DNS helped in the data interpretation. ERC performed the analysis and interpreted the data. SNZ participated in the study design and helped with the data interpretation. SSB conceived the study, participated in its design, and coordinated and drafted the manuscript. MAS participated in the study design and coordination. All authors read and approved the final manuscript.
